# Spheroid Cell Aggregation Enhanced by Enzyme‐Free Ultrasound‐Detached Cells

**DOI:** 10.1002/adbi.202500092

**Published:** 2025-08-04

**Authors:** Julien van Delft, Chikahiro Imashiro, Yuta Kurashina, Makoto Hirano, Jun Homma, Shinsuke Mochizuki, Hideharu Shimozawa, Kenjiro Takemura

**Affiliations:** ^1^ Department of Mechanical Engineering Keio University 3‐14‐1 Hiyoshi Kohoku‐ku Yokohama 223‐8522 Japan; ^2^ School of Engineering University of Tokyo Tokyo 113‐8654 Japan; ^3^ Division of Advanced Mechanical Systems Engineering Institute of Engineering Tokyo University of Agriculture and Technology Tokyo 184‐8588 Japan; ^4^ Department of Pharmacy Yasuda Women's University Hiroshima 731‐0153 Japan; ^5^ Institute of Advanced Biomedical Engineering and Science Tokyo Women's Medical University Tokyo 162‐8666 Japan; ^6^ Medical Systems and Components Operations, Canon Inc. Tokyo 146‐8501 Japan

**Keywords:** enzyme‐free, spheroids, 3D aggregates, tissue engineering, ultrasound

## Abstract

Spheroids are being widely studied as potential building blocks for complex organ engineering, tools for drug screening and cancer study. However, formation time has become the bottleneck of applications due to the need for large‐scale high‐quality spheroids production. Formation time is often dominated by ECM construction and not cell aggregation. Therefore, this study focuses on the influence of ultrasound detachment replacing conventional enzyme detachment on spheroid formation processes. Thanks to cell surface protein preservation in ultrasound detachment, cell aggregation time is reduced while decreasing the formation variabilities. Moreover, it is confirmed that cells are intrinsically more capable of aggregating through enzyme‐free detachment. On top of that, transplantations into rats showed equally successful engraftment properties for enzyme‐free detached cells. Finally, the impact on the real co‐cultured spheroid application was shown to be beneficial through more localized cell groups inside of the spheroids, possibly improving therapeutic effects and vascularization. Through this study, it is proved that ultrasound detachment can replace enzyme detachment without degrading the final spheroid properties but reducing the formation time, and variability and improving robustness and cell distribution. This opens up a new range of applications for better and faster spheroid formation in numerous bioengineering applications.

## Graphical abstract

This study demonstrates that ultrasound‐based, enzyme‐free cell detachment accelerates spheroid formation by preserving surface proteins, reducing variability, and enhancing cell aggregation. Comparable engraftment and improved spatial organization in co‐cultured spheroids are observed. These findings suggest ultrasound detachment as a robust alternative for efficient, high‐quality spheroid production in tissue engineering and related biomedical applications.

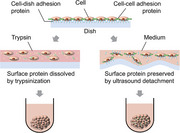

## 1 Introduction

Over the past decades, 3D cell culture processes have gained an increasing interest considering many valuable advantages over 2D cell culture. 3D cell formations have shown enhanced cell viability, proliferation activity, metabolic functions, regenerative performances and differentiation abilities.^[^ ^1^, ^2^, ^3^, ^4^ ^]^ 3D spherical cell aggregates, often called *spheroids*, are widely studied as versatile research tools in multiple fields. From being first investigated as in vitro tumors to developing cancer cures, spheroids are used today in 3D cell cultures, drug screening and as building blocks for tissue engineering. Spheroids mimic closely the sophisticated in vivo environment, enabling more efficient drug screening and more successful transplantation abilities.^[^ ^5^, ^6^, ^7^ ^]^

However, spheroids are generally required in large numbers and with low variability for tissue engineering or drug screening purposes. Spheroid‐based tissue engineering for example can require up to millions of cell aggregates depending on the objective due to intrinsic size limitations.^[^ ^8^ ^]^ To this end, the formation methods of the 3D cell aggregates have been carefully studied and developed to reduce the time cost of such studies. Initially, passive formation methods were chosen for simplicity such as the hanging drop or the liquid overlay. Although widely used, the latter rely on gravity and cell‐cell interactions only leading to a formation time from days to weeks. Those methods are complex to be automatized and applied on a large scale for mass production as manual labor is usually required.^[^ ^3^, ^5^, ^7^ ^]^ On the other hand, active methods have gained interest recently. By applying external forces on the cells, the aggregation is forced and the shape can be controlled precisely. Using acoustic, electrical, magnetic or microfluidics, thousands of cell aggregates can be formed in as short as 1 h.^[^ ^9^, ^10^, ^11^, ^12^, ^13^, ^14^ ^]^ Although those methods are promising, further culture is required to obtain well‐developed mature spheroids with highly connected extracellular matrix (ECM) from loose cell aggregates.^[^ ^3^, ^15^ ^]^ Even with a very short aggregation time, culture time cannot be greatly reduced and can be up to several days. To reduce this timespan, additives methods are usually considered. The previous literature^[^ ^16^ ^]^ supplemented mesenchymal stem cells (MSCs) with cell‐secreted ECM to improve the proliferation, survival and therapeutic effects of the formed spheroids. Hydrogels or other biocompatible materials have also been studied as ways to improve the formation of spheroids thanks to their high compatibility with ECM.^[^ ^17^ ^]^ However, those methods require additional components that may have altered effects on the cell functions, increasing the cost and manual labor required to generate the cell aggregates. As cell aggregation is driven by cell surface protein interactions, particularly based on integrins and cadherins,^[^ ^18^ ^]^ another approach could be to increase the protein concentrations in the cells that will aggregate. To this end, it was proven that cell culture, particularly the cell detachment method is decisive,^[^ ^19^ ^]^ as maintaining cell quality remains a common challenge in all spheroid formation methods. Conventionally, enzyme detachment is preferred for easiness and reliability. However, enzymes damage cell proteins in the detachment process. While this gold standard lacks alternative detachment methods, ultrasound enzyme‐free cell detachment method shows potential as a viable alternative. Through ultrasound enzyme‐free cell detachment, cell surface protein can be preserved in the vast majority and the damages to the cells are minimal.^[^ ^20^ ^]^

To study the influence of the cell protein concentration on the spheroid formation process, cells detached by ultrasound are compared with enzyme‐detached cells in the cell aggregation process in mono and co‐cultured environments through several experiments. These experiments are conducted to demonstrate the impacts of ultrasound detachment over enzyme detachment. To ensure the stability and precision of the ultrasound enzyme‐free cell detachment method for reliable comparisons across experiments, we developed a novel device capable of detaching cells using ultrasonic resonance vibrations, building upon the findings presented in ref. [20].

## 2 Result and Discussion

**Figure 1:**
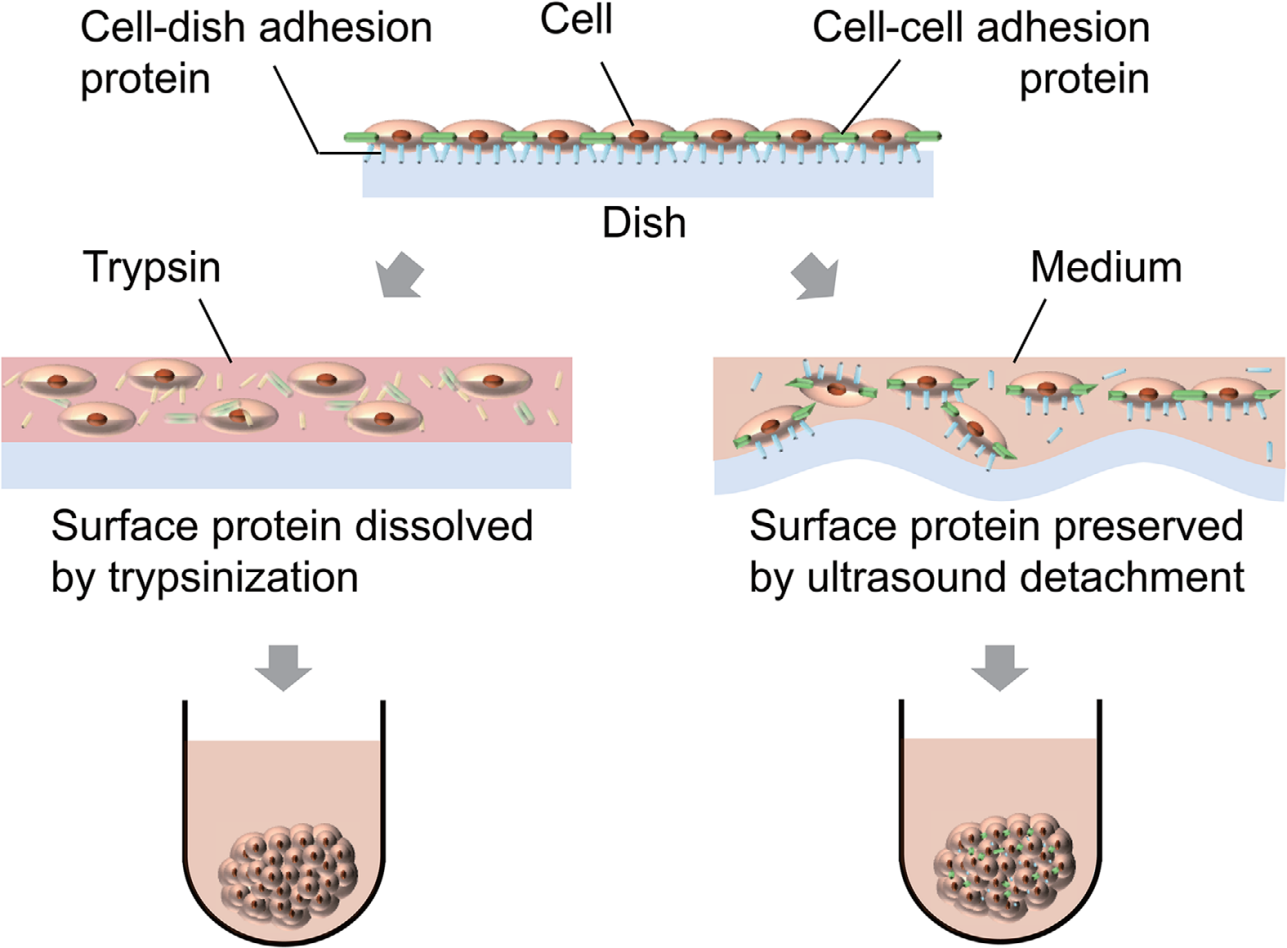
Concept of this study. Cells detached by trypsin (left) and ultrasound (right) are compared in the spheroid formation process.

**Figure 2:**
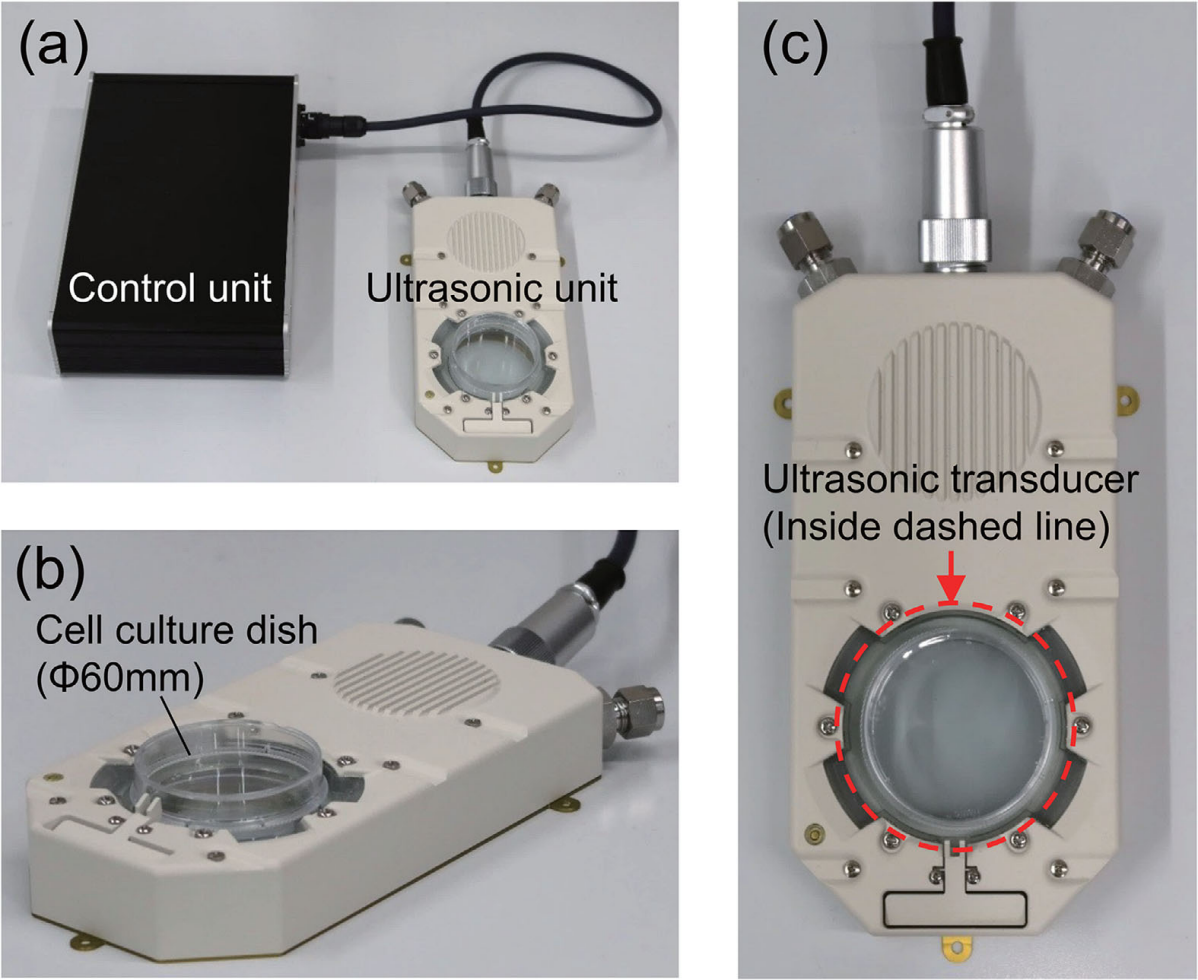
Ultrasound detachment device. a) Overall view of the detachment device from above with control unit and ultrasonic unit. b) Bird view of the ultrasonic unit with 60‐mm dish. c) Upper view of ultrasonic unit showing the ultrasonic transducer at the bottom of the 60‐mm dish.

The ultrasound cell detachment is first introduced to a spheroid formation protocol in this study as illustrated in **Figure** **1**. Cells are detached either by trypsin or using the ultrasound detachment device (**Figure** **2**) fabricated by *Canon Inc*. based on the previous research.^[^ ^20^ ^]^ Using ultrasound detachment, the cells are detached without any enzyme in the corresponding medium. Then, spheroids are formed and the influence of the detachment method on the aggregate formation is evaluated. First, mono‐cultured spheroids are fabricated conventionally and studied with comparative and time‐lapse experiments. Afterward, applications of the ultrasound detachment are proposed with transplantation, fetal bovine serum (FBS)‐free and co‐cultured applications.

### 2.1 Mono‐Cultured Comparative Spheroid Formation

**Figure 3:**
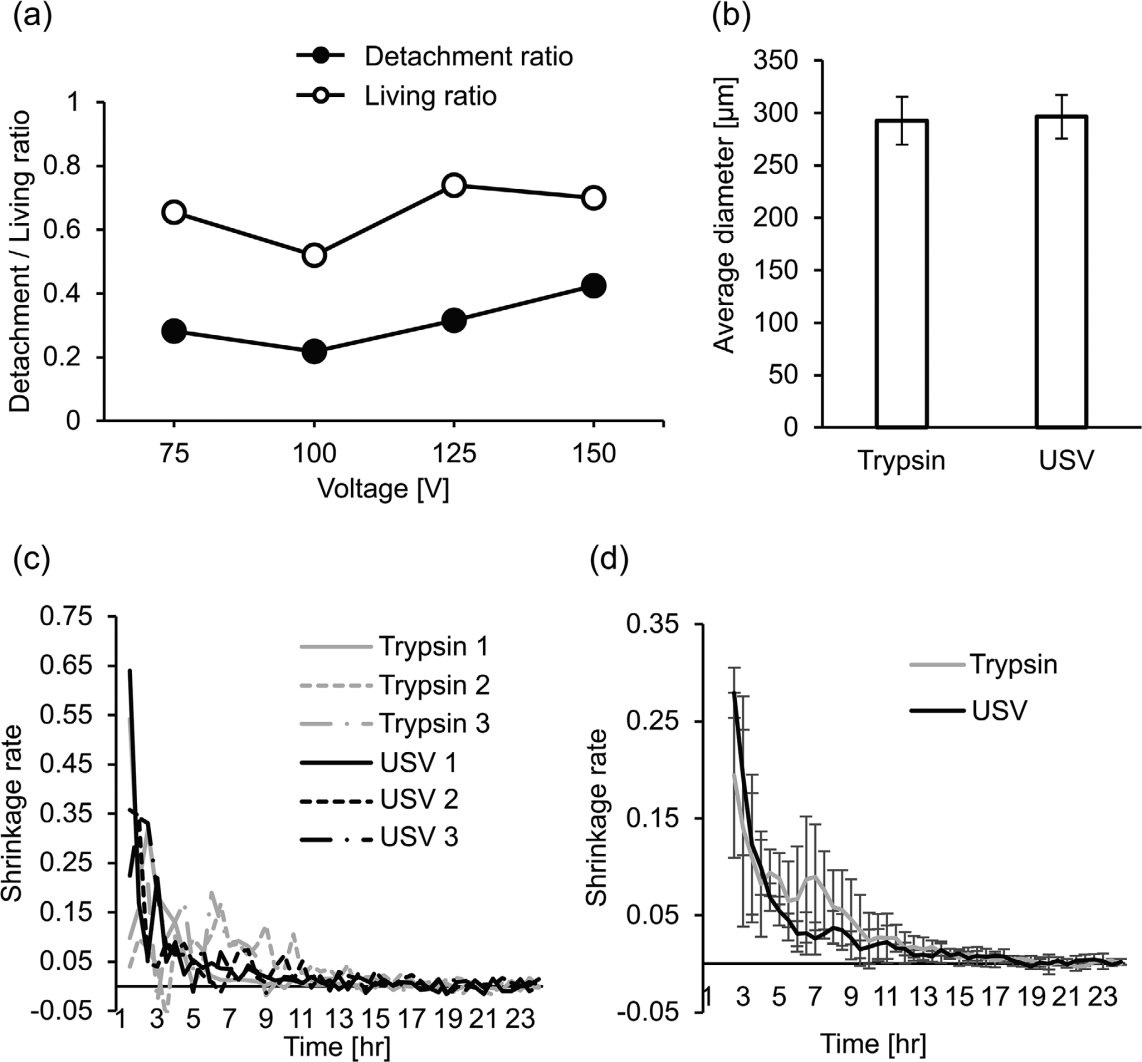
Mono‐cultured spheroid formation. a) Detachment ratio and living ratio evolutions with applied voltage (*n* = 3). b) Average diameters of spheroids formed by liquid overlay after 48 h (means ± SD, *n* = 3 with 8 spheroids each). c) Shrinkage rate evolution in the first 24 h of the formation. d) Smoothed average shrinkage rate in the first 24 h for trypsin and ultrasound detachment (means ± SD, *n* = 3). (n.s.: p ⩾ 0.05, analyzed by Student's *t*‐test).

Cultured C2C12 myoblasts (RCB0987; RIKEN Bioresource Center, Ibaraki, Japan) were detached by trypsinization and by ultrasound detachment after 2 days of culture in a 60‐mm dish (3010‐060, AGC Techno Glass Co., Ltd., Shizuoka, Japan). The ratio of detached cells and remaining cells can be seen in Figure 3a. The detachment is the highest at 42% at 150 V while keeping the living ratio at around 70%. This detachment voltage value was used in the following spheroid formation experiments.

**Figure 4:**
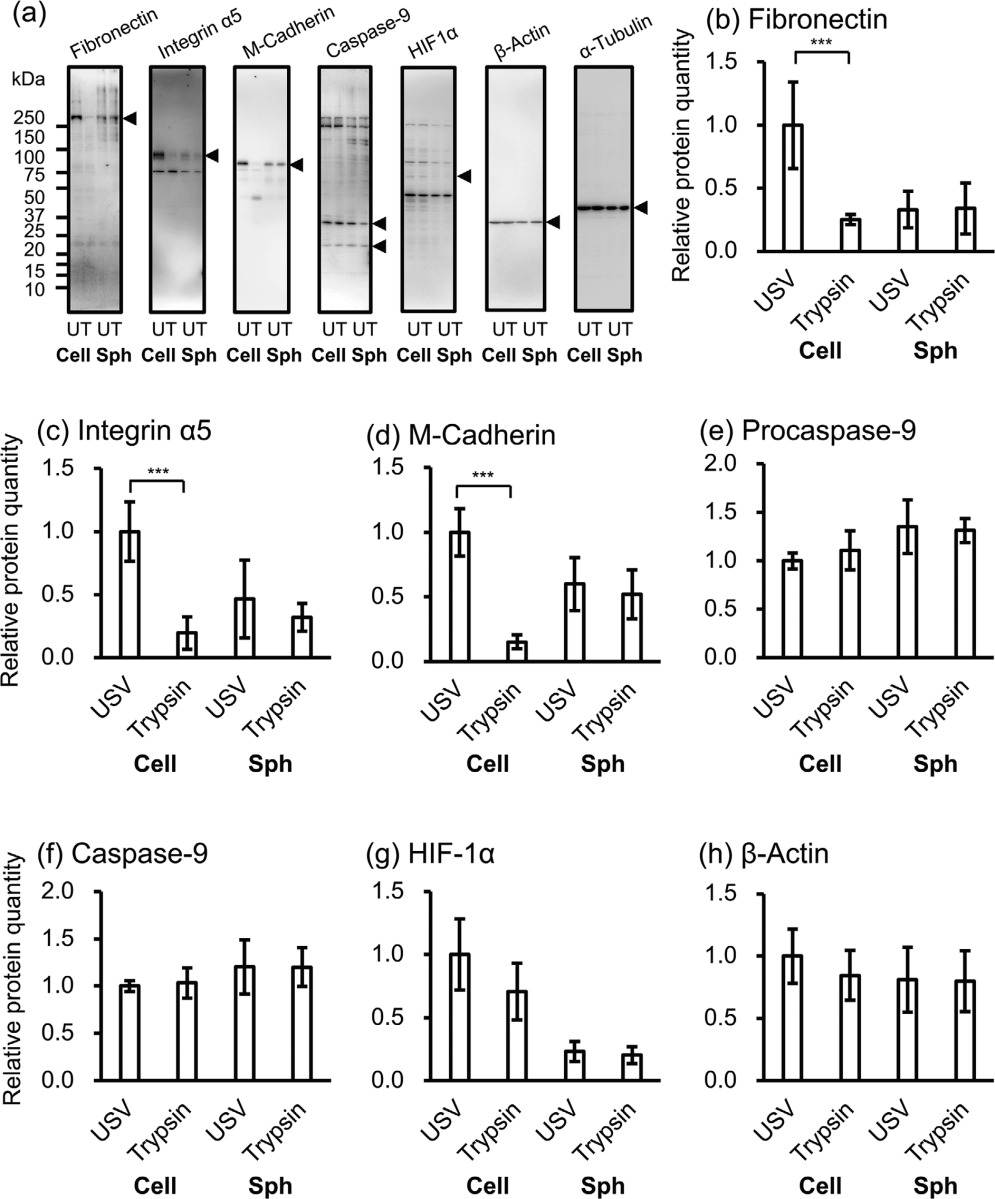
Comparison of protein quantity in the detached cells (Cell) and the spheroids (Sph). a) The detached cells by USV (U) and by trypsin (T), and the spheroids were lysed in SDS‐PAGE sample buffer. The proteins were analyzed by western blotting with the following antibodies: anti‐fibronectin (left panel), anti‐integrin α5 (second panel), anti‐M‐cadherin (third panel), anti‐caspase‐9 (center panel), anti‐hypoxia‐inducible factor‐1α (HIF‐1α; fifth panel), anti‐β‐actin (sixth panel), and anti‐α‐tubulin (seventh panel). Arrowheads indicate target bands. Anti‐caspase‐9 antibodies could detect both procaspase‐9 and activated caspase‐9 indicated by upper and lower arrowheads, respectively. Note that, because of the low signals of HIF‐1α in general western blotting, the signals were amplified using a tyramide‐signal amplification method (fifth panel). Relative protein quantities of b) fibronectin, c) integrin α5, d) M‐cadherin, e) procaspase‐9, f) activated caspase‐9, g) signal amplified HIF‐1α, and h) β‐actin were measured using their band densities on western blots. Protein quantities were normalized to the band density of α‐tubulin and expressed as the quantity relative to the USV‐detached cells (means ± SD, *n* = 4). (***: *p* < 0.005, analyzed by ANOVA).

Figure 3b illustrates the average size of the spheroids after 48 h depending on the detachment method (*n* = 3). After 48 h of culture, the sizes of spheroids did not show statistically significant differences based on the detachment method, as analyzed using Student's *t*‐test with a significance level of 5%. The cells detached by trypsinization formed spheroids of around 292.6 ± 22.9 µm and the ultrasound‐detached cells about 296.5 ± 20.7 µm in diameter.

Figure 3c shows the shrinkage rate evolution for three different measures for both trypsin and ultrasound detachment in the first 24 h of the formation. Figure 3d illustrates the averaged values of the shrinkage rate (*n* = 3). No statistically significant differences were observed, as analyzed using Student's *t*‐test with a significance level of 5%. The average spheroid formation stage timespans are displayed in **Table** **1**.

**Table 1:** Spheroid aggregation and compaction duration from shrinkage values (*n* = 3).

Detachment	Aggregation	Compaction
	duration [h]	duration [h]
Trypsin	12.33	5.33
Ultrasound	9.5	8

### 2.2 Protein Quantification

To assess the differences in the formed spheroid, the protein expressions of detached cells and formed spheroids were evaluated. We quantified the following proteins using Western blotting analysis: an ECM protein, fibronectin, which regulates α5β1 integrin mediated cell cohesion;^[^ ^21^ ^]^ adhesion molecules, integrin α5, which promotes cell aggregation with β1 integrin,^[^ ^22^ ^]^ and M‐cadherin, which is expressed at a higher level in C2C12 cell culture on the gelatin‐laminin hydrogel;^[^ ^23^ ^]^ an apoptosis‐related protein, (pro)caspase‐9, which is an activating enzyme for caspase‐3 induced at the perinecrotic area through spheroid formation;^[^ ^24^ ^]^ a hypoxia‐inducible factor, HIF‐1α, which is induced in a larger size of spheroid;^[^ ^25^ ^]^ and a cytoskeleton protein, β‐actin, which is indispensable for spheroid formation because the blocking of actin polymerization reduced the cell aggregation.^[^ ^18^ ^]^ **Figure** **4** shows the measured expression of those proteins in both detached cells and two‐day‐old spheroids (*n* = 4). The concentration of adhesion‐related proteins, fibronectin, integrin α5 and M‐cadherin were significantly higher for ultrasound‐detached cells. This result is consistent with findings from previous study.^[^ ^20^ ^]^ However, the differences in detached cells were not replicated in spheroids. No statistically relevant difference was detected in spheroids based on the detachment method.

**Figure 5:**
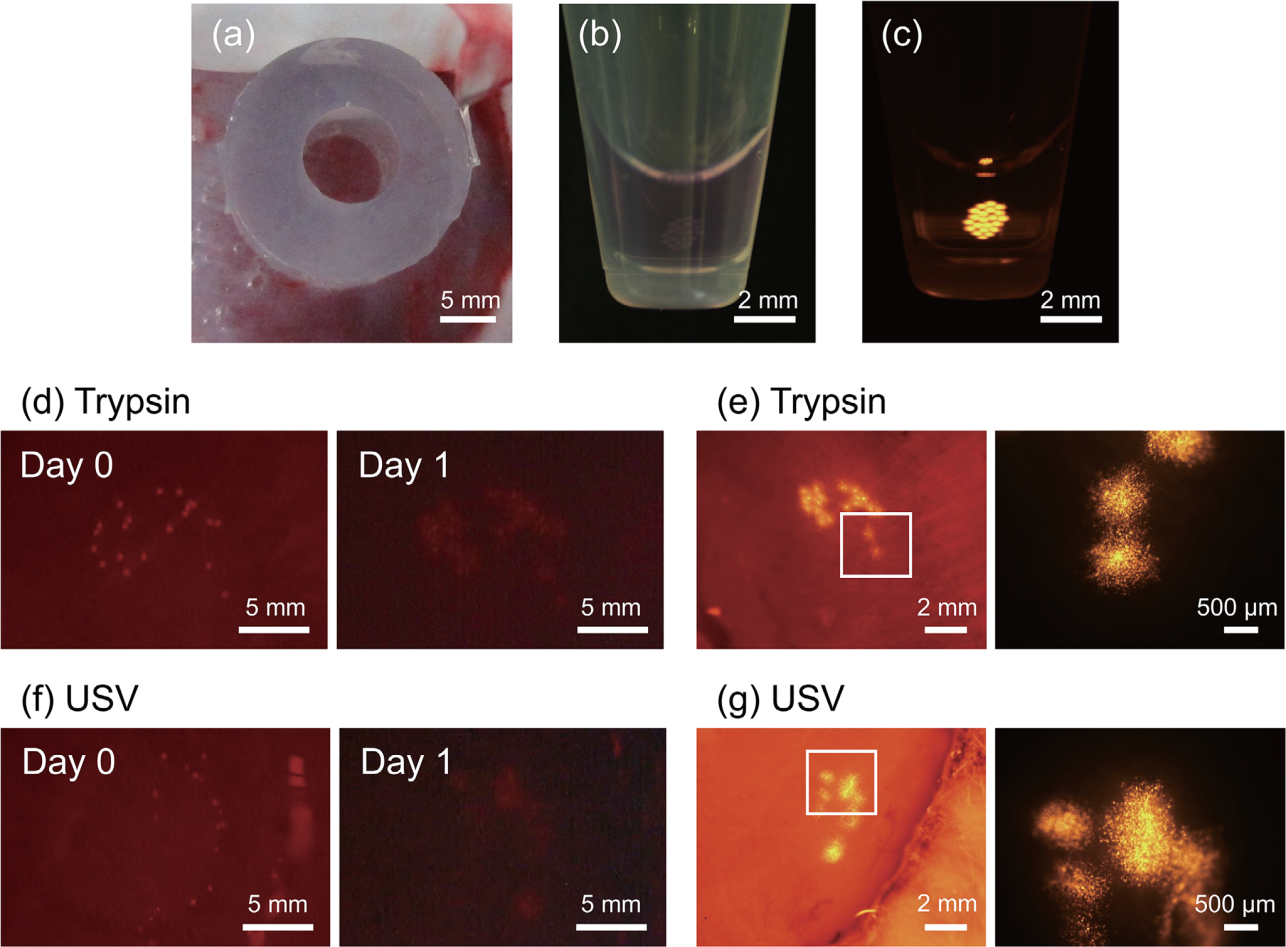
Spheroid engraftment. a) Image showing a silicone ring for spheroids transplantation set on the superficial layer of the recipient rat muscle. b,c) Images of a suspension of spheroids in a microtube captured with a stereomicroscope in (b) and with a fluorescence stereomicroscope in (c). d,f) Representative fluorescence images of transplanted spheroids captured with a surgical stereomicroscope. Red fluorescent regions indicate live‐cell labeled spheroids. Transplantation of spheroids made from cells detached by trypsin are showed in (d) and by ultrasound in (f). The left panel figure is right after transplantation, and the right panel is 1 day after transplantation. e,g) Representative images of transplanted spheroids 1 day after transplantation captured with a fluorescence stereomicroscope. Orange fluorescent regions indicate live‐cell labeled spheroids. Each right panel showing the magnified image of the white square in the left panel.

### 2.3 Transplantation Results

To evaluate the functionality of spheroids made from cells detached by trypsin and ultrasound, spheroids were transplanted on fibrin gel and on rat muscle. After being engrafted on fibrin gel, the spheroids were observed using time‐lapse live cell imaging for 25 h. The observations revealed that the spheroids made from trypsin‐detached cells flattened and the constituent cells migrated in concentric circular patterns (Video S1 and Figure S1a,b, Supporting Information). These changes in engrafted spheroids were consistent with the results observed in spheroids made from ultrasound‐detached cells (Video S2 and Figure S1c,d, Supporting Information). Subsequently, in an in vivo transplantation study, the spheroids made from ultrasound‐detached cells were consistently engrafted as follow. Transplanted spheroids were observed to engraft in the same region immediately after transplantation and one day later (Figure S1d, Supporting Information). Moreover, one day after transplantation, the spheroids flattened and the constituent cells migrated outwards, resembling the process observed in fibrin gel engraftment (**Figure** **5**f). These process of spheroids engraftment were also consistent with the spheroids made from trypsin‐detached cells (Figure 5d,e). In conclusion, spheroids made from ultrasound‐detached cells had equivalent functionality to trypsin‐detached cells regarding engraftment and cell migration.

### 2.4 FBS‐Free Spheroids

**Figure 6:**
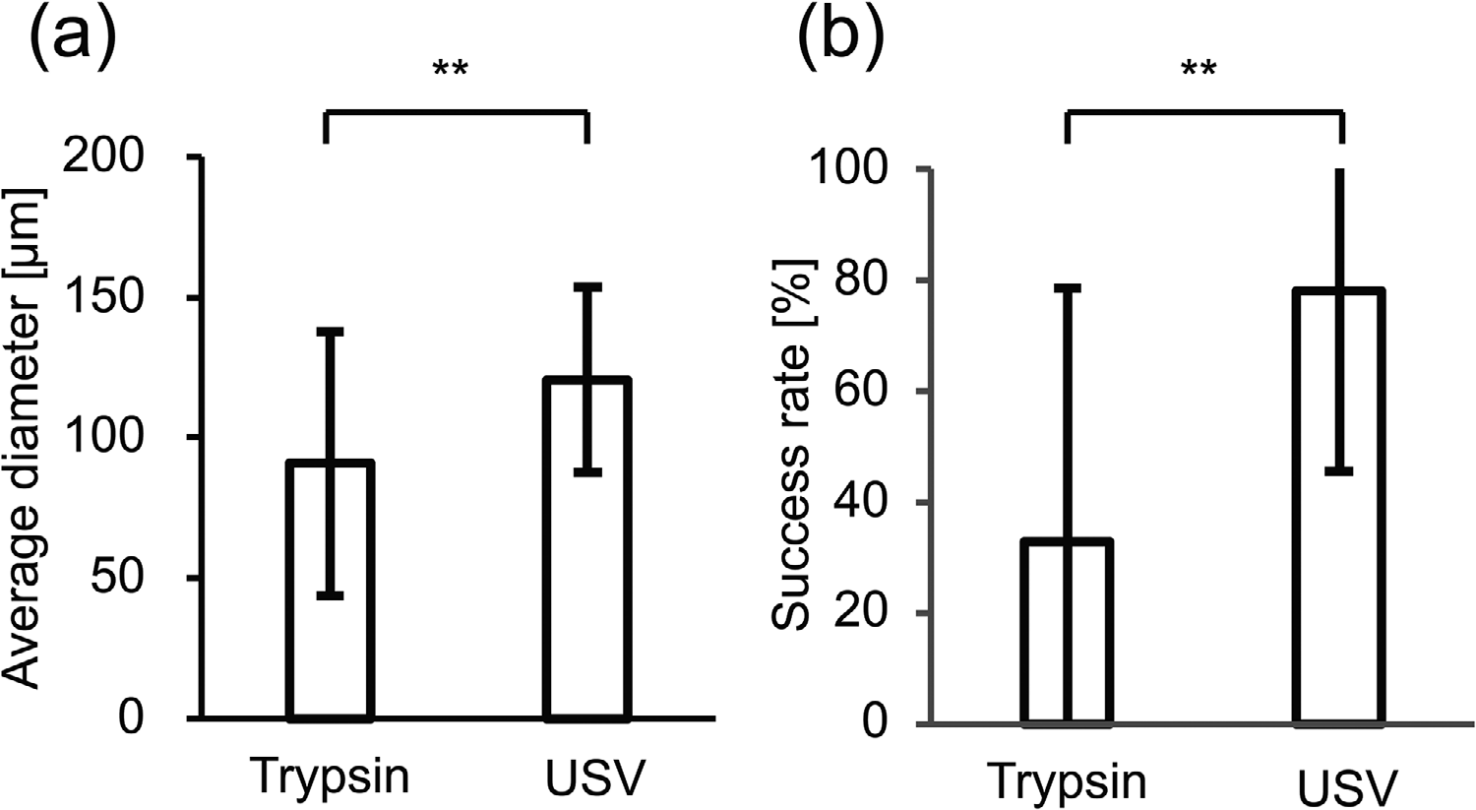
Spheroid formation in an FBS‐free medium. a) Average sizes of the spheroids formed after 48 h in an FBS‐free medium (means ± SD, *n* = 6 with 8 spheroids each). b) Percentage of aggregate bigger than 100 µm in diameter after 48 h of formation based on the detachment method (means ± SD, *n* = 6 with 8 spheroids each). (**: *p* < 0.01, analyzed by Student's *t*‐test).

Figure **6**a shows the average size of cell aggregates formed in an FBS‐free medium after 48 h (*n* = 6). In Figure 6b, the success rate is displayed for the corresponding formations (*n* = 6). It can be seen that ultrasound‐detached cells formed significantly bigger cell aggregates than trypsin‐detached cells. The average diameter of spheroids made from trypsin‐detached cells was about 90.9 ± 47.0 µm and 120.7 ± 33.2 µm from ultrasound‐detached cells. Therefore, the formation success rates were much higher for ultrasound detachment. The success for trypsin detachment was around 32.8 ± 45.8% and 78.1 ± 32.6% for ultrasound detachment.

### 2.5 Co‐Cultured Spheroids

**Figure 7:**
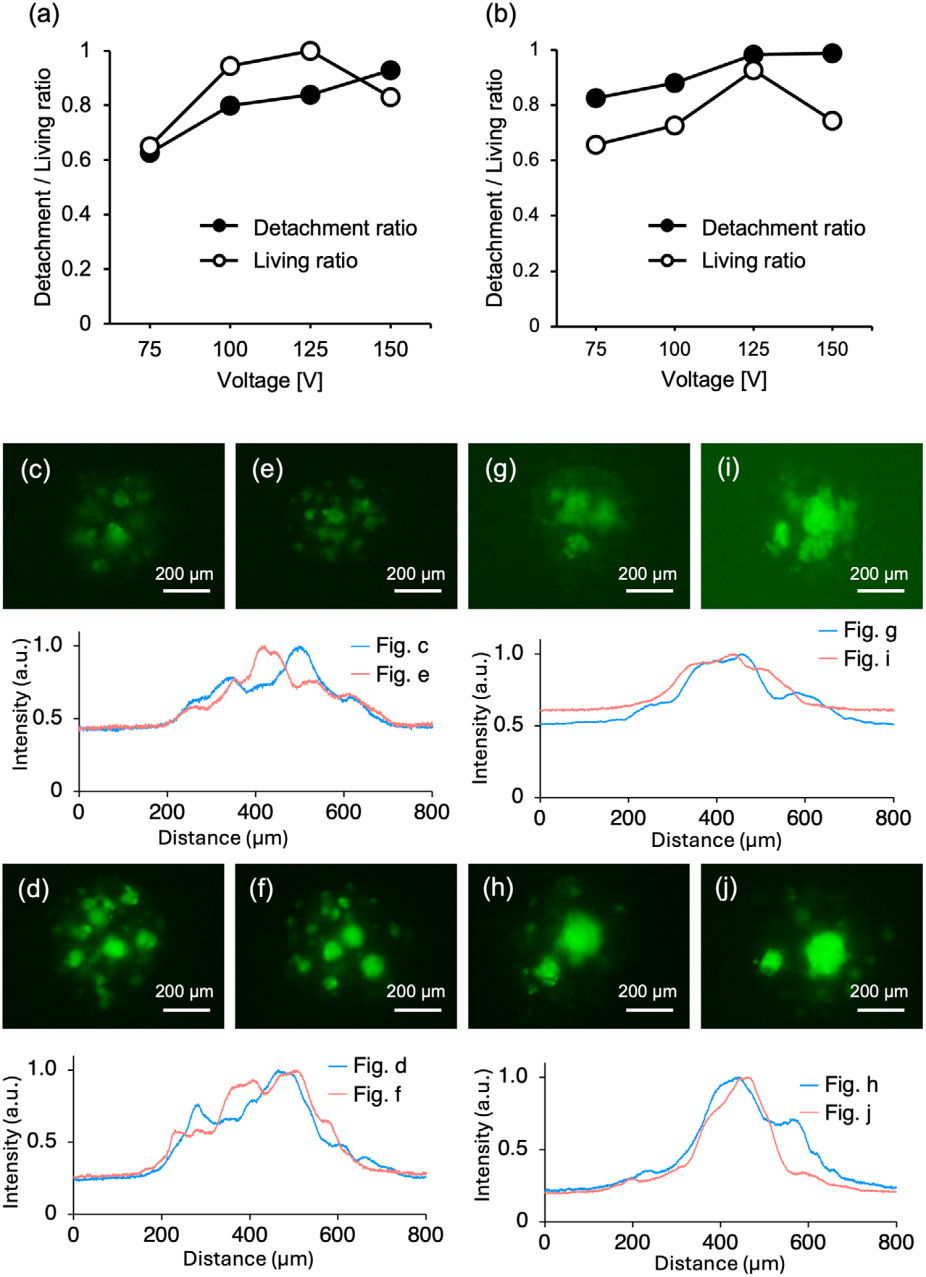
Co‐cultured spheroid formation with GFP‐HUVEC and iGL cells. a) Detachment ratio and living ratio evolutions with applied voltage for GFP‐HUVEC (*n* = 2) and b) iGL cells (*n* = 3). c,e) Spheroid made from trypsin‐detached cells after 24 h and d,f) 48 h. g,i) Spheroid made from ultrasound‐detached cells after 24 h and h,j) 48 h. The relative intensity distribution within an 800‐µm square for each condition is displayed below the corresponding fluorescent images, with the x‐axis representing the vertical position in the images.

Figure **7**a,b represents the evolution of the detachment and living ratios based on the detachment voltage. The detachment is the highest while not hurting the cells at 125 V with 84% detachment and 100% living ratio for GFP‐HUVEC (GFP‐HUVEC, cAP‐0001GFP, ANGIO‐PROTEOMIE, Boston, USA). For iGL cells (IGL01C, Cosmo Bio, Sapporo, Japan), the detachment is the highest while not hurting the cells at 125V with 98% detachment and 92% living ratio. These detachment voltage values were used in the following.

The cell distributions based on the detachment methods after 24 and 48 h are shown in Figure 7c–j. Even co‐cultured with iGL cells, only GFP‐HUVEC cells are visible under the inverted fluorescent microscope in green. The relative intensity distributions within an 800‐µm square are shown directly below the corresponding fluorescent images, with the x‐axis representing the vertical position. Comparing the spheroids made from trypsin‐detached cells after 24 h (c, e) with those made from ultrasound‐detached cells (g, i), the former exhibit a multimodal distribution, whereas the latter display a unimodal distribution. After 48 h, no clear differences are observed in the spheroids made from trypsin‐detached cells (d, f). In contrast, spheroids from ultrasound‐detached cells after 48 h (h, j) exhibit a sharper distribution. This indicates that bigger GFP‐HUVEC aggregates can be seen inside of the spheroids made from GFP‐HUVEC and iGL cells detached by ultrasound (Figure 7g–j).

### 2.6 Discussion

In this study, the influence of the detachment method and cell protein preservation on spheroid formation was studied. From our experiments, it was proven that ultrasound detachment is a valid replacement for enzyme detachment for spheroid formation. To the best of our knowledge, it is also the first proven application of ultrasound‐detached cells which opens a range of applications for enzyme‐free tissue engineering and drug testing among others.

We investigated variations in spheroid formation based on the detachment method. First, we confirmed the results found in ref. [20] by western blotting for single cells (Figure 4b–d). It was shown that ultrasound detachment leads to 5–10 times higher cell protein concentrations thanks to the cell surface protein preservation. As the preserved proteins, namely fibronectin, integrin α5 and cadherin, play a crucial role in the aggregation and compaction of spheroids, the impact of the higher starting concentrations was studied. From common knowledge,^[^ ^18^ ^]^ the cell‐cell adhesion and ECM‐cell adhesion proteins are secreted rapidly in the formation process, usually in the first 24 h. Indeed, with mono‐cultured spheroids using conventional protocols, no statistical difference could be found in the average diameter of spheroids after two days of culture (Figure 3b). The average size was around 292.6 µm for trypsin detachment and 296.5 µm for ultrasound‐detached cells. Consequently, these results suggest that the detachment method does not impact the final cell aggregate size after two days. It can be concluded that spheroids could be normalized quickly after the compaction phase. The western blotting for spheroids confirms the latter assumption as no statistically relevant differences were found in spheroids formed after two days based on the detachment method. Accordingly, transplantation into rats showed no degradation in engraftment properties. Figure 5d–g showed no degradation in engraftment and cell migration properties based on the detachment method both on fibrin gel and on rat muscle. Thus, effectively proving that our method is promising even for biomedical applications. In conclusion, ultrasound detachment can be used alternatively to conventional enzyme detachment without impacting the quality of the final produced spheroid.

On this basis, the advantages of ultrasound detachment over enzyme detachment are presumably shown in the first stages of cell aggregation. Time‐lapse experiments were preferred to precisely monitor the differences in formation speed. First, ultrasound‐detached cells produced more consistent results (Figure 3c,d). The natural variations of the trypsin state for trypsinization likely explain these variations as trypsin activity decays with storage time.^[^ ^26^ ^]^ Ultrasound detachment led to a lower variability as mechanical stresses do not change significantly between detachments. The first few hours of the time‐lapses exhibited a bigger decrease in the cell aggregate areas for ultrasound detachment, corresponding to a faster decrease in the shrinkage rate. This indicates that ultrasound detachment method leads to quicker cell aggregation in the first few hours. In all likelihood, the initial higher cell protein concentration led to fewer required protein secretions before enabling cell aggregation. Accordingly, one advantage of cell protein preservation linked to our detachment method consists of more biologically active resulting cells, possibly reducing the application time as shown in previous research.^[^ ^20^ ^]^ Interestingly, the faster aggregation is compensated by a longer compaction phase. Ultrasound‐detached cells went through 9.5 h of aggregation and 8 h of compaction on average (*n* = 3) as opposed to 12.33 h and 5.33 h for trypsin. The longer compaction duration could lead to a stronger or more developed ECM inside of the spheroids after the compaction phase.

In addition to the faster spheroid formation, cell protein preservation has direct application through the reduction of the dependence on the cell's protein production. To evaluate the intrinsic cell capacity to form aggregates predominantly through the remaining cell surface proteins, cell aggregation in an FBS‐free medium has been observed. The spheroids formed by ultrasound‐detached cells were on average 30 µm bigger in diameter or an increase of almost 33%. The latter result is largely explained by the higher surface protein concentration in ultrasound‐detached cells allowing them to form bigger cell aggregates. Similarly, the co‐cultured spheroids exhibited a more concentrated cell distribution of GFP‐HUVEC from ultrasound detachment. Two main explanations for this are considered. First, the ECM and surface proteins were retained thanks to ultrasound detachment, leading to a quick aggregate of same‐cell species inside of the spheroid. Then, ultrasound detachment usually detaches both single cells and cell blocks. The cell blocks keep their entire 2D cell–cell network which stays bound together inside of the spheroid. More localized cell groups inside the spheroid could have a beneficial effect on vascularization and therapeutic benefits.

This advancement—attributable to the preservation of cell surface proteins—has significant implications not only for the spheroid formation process but also for broader applications in regenerative medicine. From a clinical standpoint, xeno‐free media are highly recommended for in vitro cell culture and are a fundamental requirement for spheroid‐based strategies in regenerative medicine.^[^ ^27^ ^]^ The proposed technology offers a promising solution to this challenge. In addition, in standard co‐culture systems involving GFP‐HUVECs and iGL cells, mesenchymal stem cells (MSCs) are typically incorporated as adherent agents due to their active secretion of cadherins, integrins, and extracellular matrix components.^[^ ^28^ ^]^ However, in the absence of MSCs, co‐culture spheroids formed from trypsin‐detached cells exhibited a loosely aggregated structure, as shown in Figure 7c,e (24 h) and Figure 7d,f (48 h). In contrast, spheroids formed from protein‐rich, ultrasound‐detached cells demonstrated a more compact and cohesive morphology (Figure 7g,i for 24 h; Figure 7h,j for 48 h). This finding holds biological significance, as MSCs are not inherently required for iGL (pancreatic cell) spheroid formation. Therefore, the exclusion of unnecessary cell types from the co‐culture process may be achievable, offering a streamlined and optimized spheroid production strategy. This represents a notable advancement in co‐culture spheroid formation, with promising implications for the broader application of regenerative medicine. Furthermore, the feasibility of co‐culture spheroid formation in an FBS‐free environment using the proposed technology was confirmed, as shown in Figure S2 (Supporting Information). The experimental protocol mirrored that used for Figure 7, with the exception that the FBS‐free medium consisted of Dulbecco's Modified Eagle Medium/Nutrient Mixture F‐12 (DMEM/F‐12, GlutaMAX^*TM*^ supplement 10565018, Thermo Fisher Scientific Inc., Kanagawa, Japan) with 1% antibiotics (penicillin‐streptomycin 168‐23191, FUJIFILM Wako, Osaka, Japan). On Day 1, ultrasound‐detached cells formed larger aggregates compared to enzyme‐detached cells, with notably greater aggregation of GFP‐HUVECs. Interestingly, by Day 2, GFP‐HUVECs had disappeared from the aggregates formed by enzyme‐detached cells, whereas both GFP‐HUVECs and iGL cells remained present in the ultrasound‐detached aggregates. These observations warrant further investigation in future studies.

Beyond regenerative medicine, FBS‐free spheroid formation also offers significant advantages in pharmacological applications. Drugs that are efficacious in vivo typically exist in their free, unbound form, independent of serum albumin.^[^ ^29^ ^]^ Spheroids are commonly employed for drug efficacy screening; thus, generating spheroids in FBS‐free media may enhance testing precision by enabling the use of lower drug concentrations, which can reduce both toxicity and dosing requirements. Moreover, FBS can interfere with certain assays such as lactate dehydrogenase (LDH) assays, where serum‐derived background absorbance hampers measurement accuracy.

In summary, the potential demonstrated in FBS‐free spheroid formation and in eliminating the need for adherent cell types in co‐culture spheroids marks a considerable advancement. These findings carry significant therapeutic and biological implications, particularly for the fields of regenerative medicine and drug development.

In future steps, we would like to study more accurately the evolution of cell protein concentrations inside of the spheroid to quantify more precisely the impact of higher concentrations. Also, a more precise transplantation experiment is required to evaluate potential changes in the therapeutic effects thanks to the enzyme‐free processes in co‐cultured spheroids. With further investigation, ultrasonic cell detachment has the potential to contribute significantly to the automation of large‐scale spheroid production. Unlike many robotic approaches that use robotic arms to replicate the manual procedures performed by culture technicians—often facing challenges due to the fundamental differences in the dynamics of human and robotic arms—the ultrasonic detachment method does not seek to replace human skills with robotic automation. Instead, it has introduced ultrasonic actuation technology into the detachment process, offering an efficient and innovative solution, particularly for 2D cultures.

## 3 Conclusion

In this study, we enhanced the spheroid formation process by replacing enzyme‐detachment with ultrasound‐detachment. Increasing the initial protein concentrations for spheroid formations allowed to reduce the aggregation time of up to 3 h while reducing the variability. On top of that, engraftment properties were not changed based on the detachment process, effectively proving the biomedical applicability of our method. Moreover, the cell aggregations were more robust thanks to protein preservation and cell aggregates could be better formed in an FBS‐free environment. Also, the cell distribution in co‐cultured spheroid can be modified thanks to ultrasound detachment. To the best of our knowledge, this research is the first successful application of ultrasound cell detachment which opens up new possibilities in bioengineering. In this research, we proved that ultrasound detachment can be used in a variety of applications from tissue engineering to drug screening to enhance the quality of the tissues through lower variability, shorter formation time and enzyme‐free processes.

## 4 Experimental Section

### Cell Culture

For the experiments, the mouse myoblast cell line C2C12 (RCB0987; RIKEN Bioresource Center, Ibaraki, Japan) was acquired. The myoblasts were cultured in Dulbecco's modified Eagle's medium/F12 (048‐29785, Wako, Tokyo, Japan) with 2% antibiotics (Antibiotic‐Antimycotic 15240062, Thermo Fisher Scientific K.K., Kanagawa, Japan) and 10% fetal bovine serum (CELLect Gold, MP Biomedicals, Inc., Santa Ana, CA, USA). For cell culture, cell passaging was performed by trypsinization using 0.05% trypsin‐ethylenediaminetetraacetic acid (EDTA) (25300, Life Technologies, Carlsbad, CA, USA).

For the co‐cultured application, the rat pancreatic β cell line iGL (IGL01C, Cosmo Bio, Sapporo, Japan) and the GFP‐expressing Human Umbilical vein endothelial cell line (GFP‐HUVEC, cAP‐0001GFP, ANGIO‐PROTEOMIE, Boston, USA) were also purchased. The iGL cells were cultured in iGL culture medium (IGLM, Cosmo Bio, Sapporo, Japan) and the GFP‐HUVEC in Endothelial Growth Medium 2 (EGM‐2 CC‐3162, Lonza, Tokyo, Japan). The cell passages were performed by trypsinization with 0.05% trypsin‐EDTA and trypsin (0.25 mg/mL) (ReagentPack^*TM*^ Subculture Reagents CC‐5034, Lonza, Tokyo, Japan) for iGL and GFP‐HUVEC, respectively. The experiments were conducted between passages 4 and 6 for GFP‐HUVEC cells.

All cells were cultured in a humidified 5% CO_2_ incubator at 37°C.

### Detachment Device

Figure 2 illustrates the detachment device built by *Canon Inc*. To detach the cells, a common 35 or 60‐mm dish is inserted into the ultrasonic unit filled with water that works as ultrasonic transmitter. In the device, an ultrasonic transducer is composed of a piezoelectric ring and a glass disc generating the ultrasonic wave, at a swept frequency around the resonance frequency. The cells are detached in the dish by a combination of acoustic streaming, sloshing, mechanical dish vibrations and acoustic pressure.^[^ ^20^ ^]^ To improve the detachment, the cells are washed three times with phosphate‐buffered saline (PBS) (T900, Takara Bio Inc., Shiga, Japan) for most cells or HEPES Buffered Saline Solution (HEPES‐BSS) (CC‐5022, Lonza, Tokyo, Japan) for GFP‐HUVECs.

As the adhesion strength varies with the cell line, experiments were conducted to find the correct values of input voltage for the ultrasonic transducer to maximize the detachment ratio while keeping the living rate close to its maximum value. The frequency was kept in the 22–25 kHz range with a sweep‐down to surely resonate the transducer even with a dish containing medium placed on it.

### Spheroid Formation Process

The spheroids were formed by liquid overlay, seeding cells in a 96‐well plate U‐bottomed with a cell‐repellent surface (650970, Greiner Bio‐One Co., Tokyo, Japan). The cells were seeded in 200 µL of medium and cultured for one to several days depending on the objectives. The medium was changed every two days when the culture time exceeded this period. The detailed protocol is based on available literature.^[^ ^30^ ^]^

To have comparable results, the same numbers of cells detached by ultrasound and trypsin are required. However, as the ultrasound detached cells are protein‐riched, the cells are sticky and usually not single‐cells. It is not possible to count the cells accurately using conventional cell counters. Therefore, a developed estimation method is used to approximate the cell suspension density. The cells were counted using an automated cell counter (TC20, Bio‐Rad Laboratories Inc., Tokyo, Japan) and trypan blue (Trypan Blue Solution, 0.4% 15250061, Thermo Fisher Scientific, Waltham, MA, USA). Before experiments, the total number of cells in each dish is estimated by detaching three dishes by trypsinization. Then, three dishes are detached by ultrasound detachment and the remaining cells after detachment are counted by trypsinization. The average detached cells after ultrasound irradiation can be approximated by the difference between the two values as:

(1) $$N_{det}=N_{tot}-N_{rem}$$

where, *N*_*det*_, *N*_*tot*_, and *N*_*rem*_ represent the number of detached cells, total number of cells and number of remaining cells, respectively.

Based on the previous equation, the detachment ratio is defined as:

(2) $$R_{det}=\frac{N_{det}}{N_{tot}}$$

where, *R*_*det*_ is the detachment ratio.

The latter values are used to prepare the cell suspension and seed consistent cell numbers.

### Mono‐Cultured Spheroids Formations

Cultured C2C12 myoblasts were detached by trypsinization and by ultrasound detachment after two days of culture in a 60‐mm dish (3010‐060, AGC Techno Glass Co., Ltd., Shizuoka, Japan). 1000 cells were seeded in each well, 8 in total for each detachment method, in 200 µL of culture medium. The spheroids were cultured for 48 h in a humidified 5% CO_2_ incubator at 37°C. The resulting spheroid diameters were measured under a phase contrast microscope (ECLIPSE Ti, Nikon Corp., Tokyo, Japan). The western blotting (cf. the next section) analyzed similarly generated spheroids.

For time‐lapse experiments, the well plate was maintained in a humidified 5% CO_2_ environment at 37°C for coherent results. 1000 cells detached either by trypsin or by ultrasound were seeded in 200 µL in one well and the evolution was monitored. Pictures were taken every 30 min for the first 24 h under the phase contrast microscope and the area of the aggregate was measured manually in ImageJ.^[^ ^31^ ^]^ To quantify the formation stages, a new parameter was considered as already developed in ref. [18], which is called the shrinkage rate and shows the rate of change of the aggregation area. It is defined as:

(3) $$s_{i}=\frac{A_{i-1}-A_{i}}{A_{0}*\Delta t}$$

where, *s*_*i*_, *A*_*i*_, *A*_0_, and Δ*t* represent the shrinkage rate of step *i*, the measured area of the aggregate at time step *i*, the reference measured area at time step 0 and the time interval between the measures, respectively. The time step 0 is defined as 1 h after seeding to allow all the cells to gather at the bottom of the well and be measurable.

From the values of the shrinkage rate, the different phases of the spheroid formation are deduced. The formation is going from aggregation to compaction when the three moving‐averaged shrinkage rate goes below 1% or 0.01. The value is smoothed to avoid any short‐term variation due to the 3D motion of the aggregate. The spheroid is switching to the growth phase when the smoothed value of the shrinkage rate becomes negative

### Western Blotting

The detached cells and spheroids were lysed in sodium dodecyl sulfate‐polyacrylamide gel electrophoresis (SDS‐PAGE) sample buffer (3000 cells/10 µL, 50 mM Tris‐HCl (pH 6.8)/75 mM dithiothreitol/2% SDS/10% glycerol/0.001% bromophenol blue) and heated at 70°C for 5 min. The proteins (10 µL/lane) were resolved by SDS‐PAGE (3–20% Tris‐HCl gradient gel) and were transferred onto polyvinylidene fluoride membranes. The membranes were treated with Blocking One (Nacalai Tesque, Kyoto, Japan) for 30 min with shaking to block the non‐specific binding of antibodies to the membranes.

Then, the membranes were treated with the following antibodies in 5% Blocking One/Tris‐buffered saline (TBS; pH 7.4) for 12 h with shaking: 0.8 µg/mL mouse anti‐human fibronectin monoclonal antibody (TV.1; abcam, Cambridge, UK), 500‐fold dilution of rabbit anti‐human integrin α5 polyclonal antibody (D7B7G; Cell signaling technology, Boston, MA, USA), 1000‐fold dilution of rabbit anti‐human M‐cadherin monoclonal antibody (D4B9L; Cell signaling technology), 2.0 µg/mL mouse anti‐human hypoxia‐inducible factor‐1α (HIF‐1α) monoclonal antibody (H1alpha67; Novus biologicals, Centennial, CO, USA), 1000‐fold dilution of mouse anti‐caspase‐9 monoclonal antibody (MBS767529; Mybiosource, San Diego, CA, USA), 1000‐fold dilution of rabbit anti‐mouse β‐actin polyclonal antibody (#4967; Cell signaling technology), and 1000‐fold dilution of mouse anti‐chicken α‐tubulin monoclonal antibody (DM1A; Cell signaling technology).

After washing three times with TBS containing 0.05 % Tween 20 (TBST), the membranes were treated with a 5000‐fold dilution of horseradish peroxidase (HRP)‐conjugated goat anti‐mouse IgG (12‐349; Sigma‐Aldrich, MO, USA) or anti‐rabbit IgG F(ab')2 fragment (NA9340‐1ML; Cytiva, Tokyo, Japan) for 30 min with shaking. The membranes were washed three times with TBST. For HIF‐1α detection, we used the tyramide‐signal amplification (TSA) method with TSA biotin kit (PerkinElmer, Waltham, MA, USA). After washing three times with PBS (pH 7.4), the membranes were treated with 500‐fold dilution of biotinyl‐tyramide stock solution in PBS for 15 min. After washing three times both with PBS (pH 7.4) and subsequently with TBS T, the membranes were treated with 5000‐fold dilution of HRP‐conjugated streptavidin (Invitrogen, Carlsbad, CA, USA) in 5% Blocking One/TBS and were washed with TBS T three times. All protein bands were visualized with an Immobilon Western chemiluminescent HRP substrate (Millipore, Billerica, MA, USA), scanned, and analyzed using LAS‐4000 image analyzer (Fujifilm, Tokyo, Japan). The band density was normalized with α‐tubulin band and was expressed as the quantity relative to the ultrasound‐detached cells.

### Time‐Lapse Live‐Cell Imaging of Spheroids During the First 25 Hours After Transplantation on Fibrin Gel

To monitor dynamic changes of engrafted spheroids, the spheroids were imaged from 1 to 25 h after transplantation using a time‐lapse live‐cell imaging system (LCV110, Olympus). Prior to detaching C2C12 cells from culture dishes using trypsin or ultrasound, the cells were stained with Cell Explorer Live Cell Tracking Kit Orange Fluorescence (AAT Bioquest, Sunnyvale, CA, USA). Subsequently, C2C12 spheroids were constructed as described above. The spheroids were then seeded onto a 35‐mm glass‐bottom dish (Matsunami Glass, Osaka, Japan) coated with 400 μL of fibrin gel and cultured in 2 mL of DMEM/F12 medium containing 10% FBS and 2% penicillin‐streptomycin. The method for constructing the fibrin gel was described previously.^[^ ^32^ ^]^ The dish was inserted into the incubator of a time‐lapse live‐cell imaging system (37°C, 5% CO_2_). “Orange” fluorescence images were captured every hour for 25 h. Additionally, pre‐ and post‐live‐cell imaging, phase‐contrast images were captured using a microscopy system (BZ‐9000; Keyence, Osaka, Japan).

### Transplantation of Spheroids into Athymic Rats

Male athymic rats (F344/NJcl‐rnu/rnu; CLEA Japan, Tokyo, Japan) were utilized in the study. For the transplantation procedure, rats were anesthetized with 2–4% inhaled isoflurane, and an incision was made on the dorsal skin to expose the superficial gluteal muscles. Subsequently, a silicone ring with a 7.5 mm inner diameter (Figure 5a) was placed on the muscles. C2C12 spheroids stained with the Cell Explorer Live Cell Tracking Kit Orange Fluorescence were collected in 1.5 mL microtubes (Figure 5b,c) containing 100 μL PBS, with 20 spheroids per tube. The spheroid suspension was then transplanted into the inner side of the silicone ring. After 30 min, the silicone ring was gently removed. To prevent adhesion to surrounding tissue, the transplanted spheroids were covered with a porous ethylene‐vinyl alcohol membrane (EVAL membrane; Kuraray Co., Ltd., Tokyo, Japan). Finally, the skin was sutured. The following day, the spheroids were observed under 2–4% inhaled isoflurane anesthesia. Images of the animal experiments (Figure 5d,f) were captured using a surgical stereomicroscope equipped with an image capturing system (M651, Leica Microsystems, Wetzlar, Germany; 3CCD camera, Toshiba Corp., Tokyo, Japan; and Video Capture Box, I‐O Data Device, Kanazawa, Japan). Images of the spheroids (Figure 5b,c,e,g) were captured using a fluorescence stereomicroscopy system (MVX10 and cellSens Dimension, Olympus).

### FBS‐Free Application

Cultured C2C12 myoblasts were detached by trypsinization and by ultrasound detachment after two days of culture in a 60‐mm dish (3010‐060, AGC Techno Glass Co., Ltd., Shizuoka, Japan). 1000 myoblasts were seeded in 200 µL of FBS‐free medium composed of Dulbecco's modified Eagle's medium/F12 (048‐29785, Wako, Tokyo, Japan) with 2% antibiotics (Antibiotic‐Antimycotic 15240062, Thermo Fisher Scientific K.K., Kanagawa, Japan). Cells were either detached by trypsin or ultrasound to compare the influence of the retention of proteins in spheroid formation in an FBS‐free environment. After 48 h of culture, the aggregates were measured under a phase‐contrast microscope. The formation was considered successful if the aggregate size exceeded 100 µm in diameter.

### Co‐Cultured Application

Cultured GFP‐HUVEC and iGL cells were detached by trypsinization and by ultrasound detachment after six days of culture in 60‐mm dishes (3010‐060, AGC Techno Glass Co., Ltd., Shizuoka, Japan). 2000 iGL cells were seeded with 1000 GFP‐HUVEC cells in a 2:1 ratio in 200 µL of culture medium made from Dulbecco's Modified Eagle Medium/Nutrient Mixture F‐12 (DMEM/F‐12, GlutaMAX^*TM*^ supplement 10565018, Thermo Fisher Scientific Inc., Kanagawa, Japan) with 10 % FBS (CELLect Gold, MP Biomedicals, Inc., Santa Ana, CA, USA), 1% antibiotics (Penicillin‐Streptomycin Solution 168‐23191, Wako, Tokyo, Japan) and L‐ascorbic acid 5µL/mL (L‐ascorbic acid phosphate magnesium salt n‐hydrate 013‐12061, Wako, Tokyo, Japan). The cell distributions inside of the spheroid were observed through an inverted microscope (Ti‐S30‐EDF‐Ph‐5SV, Nikon Corp., Tokyo, Japan) with a green filter (FITC, Nikon Corp., Tokyo, Japan). The medium was changed every two days.

### Ethics

All animal experiments were approved by the Ethics Committee for Animal Experimentation of Tokyo Women's Medical University (Approval number AE23‐150) and performed according to the Guidelines of Tokyo Women's Medical University on Animal Use and complied with the ARRIVE guidelines for the care and use of laboratory animals. All animals were housed in individual cages and maintained at a constant temperature and humidity under a 12 h light cycle. Animals were given free access to food and water. Animal euthanasia was performed by exsanguination under 5% isoflurane in accordance with the American Veterinary Medical Association euthanasia guidelines.

### Statistical Analysis

In Western blotting, protein band intensities were normalized to the α‐tubulin band and expressed relative to those in ultrasound‐detached cells. For other experiments, raw data were used for statistical analyses. Values are presented as means ± standard deviations (SD). Sample sizes varied by experiment and are specified in the legends of Figures 3, 4, 6, 7. Statistical significance was evaluated using Student's *t*‐test for comparisons between two groups, and one‐way ANOVA for comparisons among three or more groups, performed using R version 4.2.1. A *p* value less than 0.05 was considered statistically significant.

## Conflict of Interest

The authors declare no conflict of interest.

## Supporting information

Figure S1

Figure S2

Video S1

Video S2

## Data Availability

The data that support the findings of this study are available from the corresponding author upon reasonable request.
