# Supplementary material for: Spheroid Cell Aggregation Enhanced by Enzyme‐Free Ultrasound‐Detached Cells
Source: Adv Biol (Weinh). 2025 Aug 4;9(8):e00092. doi: 10.1002/adbi.202500092 (PMC12365725; doi:10.1002/adbi.202500092)
Supplement: Supplementary file 1 — Figure S1 [file ADBI-9-e00092-s001.pdf]

ADVANCED BIOLOGY

Supporting Information

for *Adv. Biology*, DOI 10.1002/adbi.202500092

Spheroid Cell Aggregation Enhanced by Enzyme-Free Ultrasound-Detached Cells

*Julien van Delft, Chikahiro Imashiro, Yuta Kurashina, Makoto Hirano, Jun Homma, Shinsuke Mochizuki, Hideharu Shimozawa and Kenjiro Takemura**

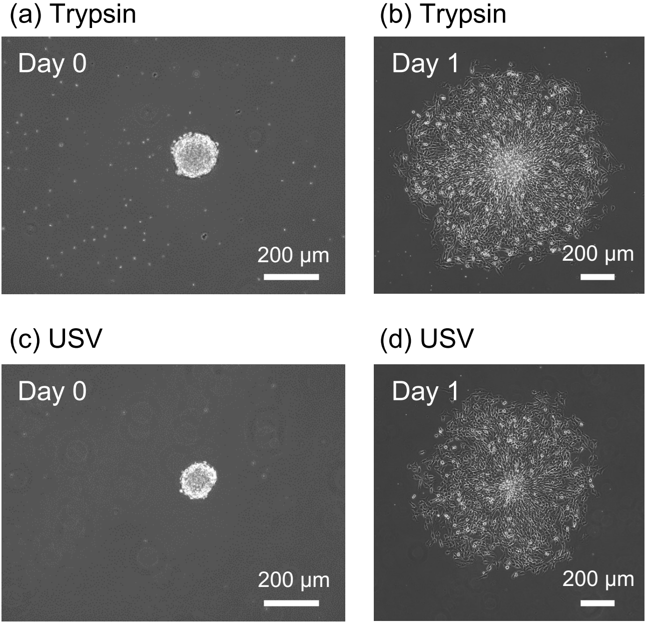

Supplementary Figure 1. Engraftment of the spheroids made from (a)(b) trypsin-detached cells and (c)(d) ultrasound-detached cells on fibrin gel.
