# Supplementary material for: Spheroid Cell Aggregation Enhanced by Enzyme‐Free Ultrasound‐Detached Cells
Source: Adv Biol (Weinh). 2025 Aug 4;9(8):e00092. doi: 10.1002/adbi.202500092 (PMC12365725; doi:10.1002/adbi.202500092)
Supplement: Supplementary file 2 — Figure S2 [file ADBI-9-e00092-s002.pdf]

ADVANCED BIOLOGY

Supporting Information

for *Adv. Biology*, DOI 10.1002/adbi.202500092

Spheroid Cell Aggregation Enhanced by Enzyme-Free Ultrasound-Detached Cells

*Julien van Delft, Chikahiro Imashiro, Yuta Kurashina, Makoto Hirano, Jun Homma, Shinsuke Mochizuki, Hideharu Shimozawa and Kenjiro Takemura**

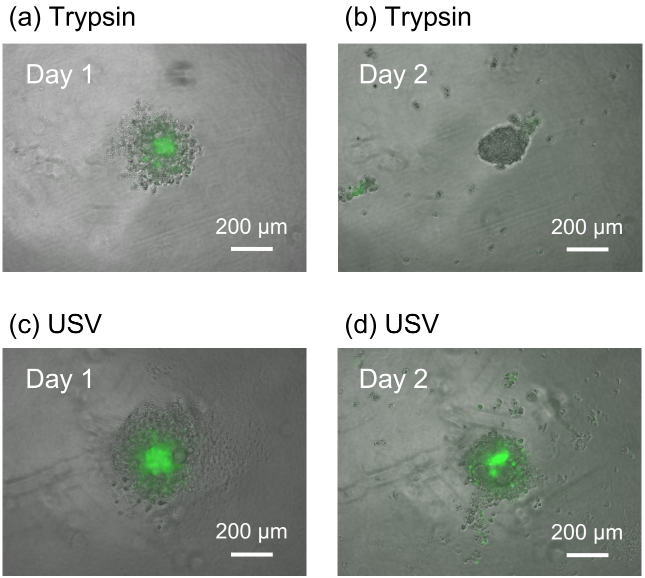

Supplementary Figure 2. Co-culture spheroid formation using GFP-HUVEC (green) and iGL cells in an FBS-free medium, presented with overlayed phase-contrast and fluorescence images. (a)(b) Trypsin-detached cells and (c)(d) ultrasound-detached cells. Cultured GFP-HUVEC and iGL cells were detached by trypsinization and by ultrasound detachment after culturing iGL cells for 6 days and GFP-HUVEC cells for 3 days in 60-mm dishes (3010-060, AGC Techno Glass Co., Ltd., Shizuoka, Japan). 2,000 iGL cells were seeded with 1,000 GFP-HUVEC cells in a 2:1 ratio in 200 μL of FBS-free medium composed of Dulbecco's Modified Eagle Medium/Nutrient Mixture F-12 (DMEM/F-12, GlutaMAX™ supplement 10565018, Thermo Fisher Scientific Inc., Kanagawa, Japan) with 1% antibiotics (penicillin-streptomycin 168-23191, FUJIFILM Wako, Osaka, Japan). Phase-contrast and fluorescence images of co-cultured spheroids were captured using a BZ-X800 fluorescence microscope (Keyence Corporation, Osaka, Japan).
